# NK Cells in a *Tug-of-War* With Cancer: The Roles of Transcription Factors and Cytoskeleton

**DOI:** 10.3389/fimmu.2021.734551

**Published:** 2021-09-14

**Authors:** E Hui Clarissa Lee, Darren Chen Pei Wong, Jeak Ling Ding

**Affiliations:** Department of Biological Sciences, National University of Singapore, Singapore, Singapore

**Keywords:** natural killer cells (NK), cancer, tumour microenvironment (TME), NK exhaustion, NK cell receptors, T-box transcription factors, cytoskeletal dynamics, mechanotransduction

## Abstract

Natural killer (NK) cells are innate immune cells which play a key role in shaping the immune response against cancer. Initially hailed for their potential to recognise and eliminate tumour cells, their application has been greatly hindered by the immunosuppressive tumour microenvironment (TME) which suppresses NK functions (e.g., cytotoxicity). This dysfunctional state that is accompanied by phenotypic changes such as upregulation of inhibitory receptors and downregulation of activating receptors, forms the basis of what many researchers have referred to as ‘exhausted’ NK cells. However, there is no consensus on whether these phenotypes are sufficient to define an exhausted state of the NK cell. While recent advances in checkpoint inhibition appear to show promise in early-stage pre-clinical studies, much remains to be fully explored and understood in the context of the TME. The TME is where the NK cells are subjected to interaction with various cell types and soluble factors, which could exert an inhibitory effect on NK cytotoxicity. In this review, we provide an overview of the general markers of NK cell exhaustion viz, the surface activating and inhibitory receptors. We also highlight the potential role of T-box transcription factors in characterising such a dysfunctional state and discuss the often-overlooked mechanism of cell cytoskeletal dynamics in regulating NK cell function. These aspects may further contribute to NK exhaustion or NK revival in cancer and may open new avenues to explore cancer treatment strategies.

## 1. Introduction

Natural killer cells (NK) belong to the family of Group 1 Innate Lymphoid Cells (ILC), and have been well-characterised for their cytotoxic functions against a wide variety of pathogens, infections and cancers (1).

Distinct from the adaptive immune cells, NK cells are purported to recognise target cells without prior antigen sensitisation (2). Instead, classical schools of thought imply that NK cell activation depends heavily on the functional state and balance of surface activating and inhibitory receptors (2), which recognise stress-induced ligands or detect a ‘missing self’, characterised by the lack of MHC class I molecules on cancer cells or infected cells (1). These characteristics make NK cells an attractive immune sentinel for cancer immunomodulation, especially in view of the shortfalls in immunotherapy, which has thus far been largely focused on CAR-T and immune checkpoint inhibition (ICI) targeting T cells. Some of these limitations in T cell based therapy include acquired mutations in antigen presentation genes with subsequent loss of antigen presentation machinery (3), and the heterogeneous nature of cancers resulting in patient non-response or relapse (4). These issues have undermined the long-term efficacy of T cell-mediated cancer therapy and have triggered interest in exploring NK cells as a complementary, or potentially a more efficacious therapeutic option.

Despite the promising potential of NK cells, clinical trials utilizing NK cell immunotherapy have thus far yielded limited outcomes. With traditional adoptive transfer either from an allogeneic or autologous source, NK cells were observed to quickly lose their function *in vivo* (5), exhibiting what has been deemed an exhausted phenotype. This has prompted a paradigm shift in exploring strategies for restoring and sustaining NK cell functions, such as checkpoint blockade or the genetic engineering of NK cells to express exogenous activating receptors. Recent trends to investigate novel and practical ways to reverse NK cell dysfunction are anticipated to provide substantial understanding of how cancer suppresses and sustains the loss of NK cell activity.

While the dysfunctional NK cells observed in cancer patients have been described by various groups as having an ‘exhausted’ phenotype, it is worth noting that the concept of NK cell exhaustion remains unclear. Thus far, NK cell exhaustion is commonly used to refer to NK cells exhibiting reduced effector function as well as up- and down-regulation of various activating and inhibitory surface receptors, respectively. In this review, we adopt these phenotypic changes and accompanying functional impairment to be the basis of NK cell exhaustion, and we discuss how they contribute to exhaustion, and propose additional aspects that may provide a more complete picture of NK cell exhaustion. We also summarise the roles of numerous players in the tumour microenvironment (TME) which contribute to the dysfunctional state of NK cells. Importantly, various overlooked mechanisms of NK cell regulation are highlighted.

## 2. Characteristics of Dysfunctional/Exhausted NK Cells in Cancer

Functionally impaired NK cells have been observed across a wide range of solid tumours as well as haematological malignancies (6, 7) and often also show reduced infiltration into tumour sites (7, 8). Peripheral NK cells from cancer patients as well as tumour-infiltrating NK cells exhibit reduced effector functions such as (i) decreased expression of the membrane protein CD107a, which is a degranulation marker, (ii) decreased secretion of cytokines IFNγ (9, 10) and TNFα (10), and the cytotoxic molecules, perforin (11) and granzyme (12), all of which result in reduced NK cell function against tumour cells. This loss of function in NK cells is often referred to as ‘exhaustion’, a phenomenon which was first observed in effector T cells present in persistent chronic infections and cancers (13, 14). Diminished effector function and accompanying phenotypic changes have been similarly observed in both exhausted T cells and NK cells. In contrast to T cell exhaustion, which is clearly defined both functionally and phenotypically (13), no strict consensus has thus far been established on what exactly constitutes an exhausted state of NK cells. This has prompted more than a decade of research on the characterisation of NK cell exhaustion.

In addition to the often observed functional impairment, numerous studies have also suggested the imbalance in the expression levels of NK cell surface activating and inhibitory receptors to be an indicator of exhaustion (6, 15–17). A diverse repertoire of activating and inhibitory receptors is expressed on the NK cell surface, and the balance and spatial-temporal integration of signals from these receptors have been established to impact NK cell effector function (18). Inhibitory receptors include the killer cell immunoglobin receptors (KIRs), CD94/NKG2A and leukocyte immunoglobin-like receptors (LIRs), which typically function by recruitment of SHP-1/SHP-2, then inducing Vav1, LAT, and PLC*γ*1/2 dephosphorylation and Crk phosphorylation, which in turn inhibit NK cell activation signals and hence its function (19). Major activating receptors include the natural cytotoxic receptors (NCRs), NKG2D and DNAM-1, whose activating signals are typically transmitted through receptors binding to ITAM-containing adaptor proteins such as DAP10, DAP12, and FcR*γ* (19). Engagement of these NK surface receptors with their respective ligands thus results in the transduction of either activating or inhibitory signals through various signalling pathways, which eventually converge on key processes regulating NK cell cytotoxicity such as target cell conjugation, degranulation, and cytokine release (19).

Due to the sheer number of receptors present and many receptors sharing recognition of the same ligands, there is no consistent standard yet as to which of these receptors are specific and precise markers of exhaustion. However, it is clear that the altered expression of multiple receptors is key to characterising exhaustion: for example, activating receptors, with the exception of CD16, are not able to activate NK cells on their own (19). In the case of the inhibitory receptor TIGIT, it has also been reported that TIGIT expression levels on NK cell surface are variable even in healthy individuals (20). Although higher TIGIT expression on healthy human NK cells correlates with reduced cytotoxic potential (20), these NK cells still largely retain their function. Thus, the elevated expression of a particular inhibitory receptor or downregulation of a single activating receptor does not necessarily mark a severely dysfunctional NK cell state as is observed in the case of chronic infections and cancers. In other words, the altered expression of a single receptor is unlikely to significantly alter NK cell activation status, instead, multiple activating and/or inhibitory receptors have to be down- or up- regulated, respectively, to result in NK cell exhaustion.

In the context of cancer, these receptors have been implicated as markers of NK cell exhaustion, as their dysregulated expression is often associated with reduced NK antitumour activity (**Table 1**). Upregulation of inhibitory receptors and downregulation of activating receptors typically correlates with reduced NK cell function as discussed above. Existing attempts have also been made at targeting these receptors (**Table 1**) for the reinvigoration of NK cell function.

**Table 1:** Archetypal NK receptors in NK cell function, exhaustion and restoration.

NK cell Receptors	Activating/Inhibitory	Status/Modification	Context	Ref
NKG2A	Inhibitory	Upregulated	Increased expression in intratumour Hepatocellular Carcinoma (HCC) tissues from human patients, correlating with poor prognosis and functional exhaustion Increased expression in peripheral and tumour-associated NK cells in breast cancer patients	(10, 21)
		Antibody blockade	Phase II clinical trial – in combination with cetuximab, increased NK cell killing by antibody-dependent cellular cytotoxicity (ADCC) in patients with squamous cell carcinoma of head and neck	(22)
PD-1	Inhibitory	Upregulated	Increased expression on NK cells from myeloma patients (reportedly no expression on healthy donor NK cells) Increased expression on both peripheral and tumour-infiltrating NK cells from patients with digestive cancers. Poor prognosis in liver and esophageal cancers.	(23, 24)
		Antibody blockade	Expanded NK cells from healthy donor peripheral blood – increased cytotoxicity against multiple myeloma cell lines, human and murine model of Multiple Myeloma (MM) Mouse model of lymphoma – blocking antibody against PD-1 reduced tumour progression	(24–26)
TIGIT	Inhibitory	Upregulated	Increased expression on intratumoral NK cells from soft tissue sarcoma, colon and endometrial cancer patients	(27–29)
		Downregulated	Decreased expression on tumour-infiltrating NK cells from melanoma patients	(30)
		Antibody blockade^a^	Primary NK cells from sarcoma patients – increased degranulation and cytotoxicity against sarcoma cell lines Peripheral NK cells from melanoma patients – increased cytotoxicity against melanoma cell line and increased IFNγ production Mouse models of various cancers: colon, breast, fibrosarcomas – reduced tumour volume, increased CD107, TNF and IFNγ expression	(27, 28, 30)
TIM-3	Inhibitory	Upregulated	Increased expression on peripheral NK cells from melanoma and bladder cancer patients, corresponding with poor prognosis. Increased expression on intratumoral NK cells from endometrial and bladder cancer patients	(29, 31, 32)
		Downregulated	Decreased expression on healthy human NK cells upon exposure to glioblastoma cell lines, corresponding with decreased cytotoxicity and IFNγ production.	(33)
		Antibody blockade	Primary NK cells from melanoma patients and healthy donor NK cells – increased NK cell cytotoxicity against four melanoma cell lines	(31)
DNAM-1	Activating	Downregulated	Decreased expression on tumour-associated NK cells from breast and ovarian carcinoma patients Decreased expression on peripheral and tumour-associated NK cells in gastric and breast cancer patients	(10, 34, 35)
		Overexpression	NK-92 cell line – increased degranulation against primary sarcoma and various other cancer cell lines	(36)
NKG2D	Activating	Downregulated	Decreased expression on tumour-infiltrating NK cells in breast cancer and melanoma patients. Decreased expression on peripheral NK cells from melanoma, breast and gastric cancer patients.	(10, 30, 31, 35)
		Overexpression	NK-92 cell line – increased degranulation against primary sarcoma and various other cancer cell lines Primary NK cells from metastatic melanoma patients – enhanced NK cell cytotoxicity *in vitro* against target K562 cells	(36, 37)
NKp30	Activating	Downregulated	Decreased expression on peripheral NK cells from breast and gastric cancer patients and associated with cancer progression Decreased expression on tumour-associated NK cells from breast cancer patients	(10, 35)
NKp44	Activating^b^	Antibody blockade	NK-92 cell line *in vitro* – increased cytotoxic activity and IFNγ release against solid tumour and leukemia cell lines NK-92 and HNSCC patient autologous NK cells in PDX-bearing mice – increased degranulation and inhibited tumour growth	(38)
NKp46	Activating	Downregulated	Decreased expression on peripheral NK cells from gastric cancer patients and associated with cancer progression Decreased expression on peripheral NK cells from melanoma patients.	(31, 35)
		Overexpression	Mouse model of melanoma – increased NK cytotoxicity and tumour clearance	(39)

^a^TIGIT blockade alone increases IFNγ production in circulating NK cells, but has to be used in combination with IL-15 to promote increased cytotoxicity in tumour infiltrating NK cells (30). ^b^NKp44 is classified as an activating receptor, but can also have inhibitory effects when engaged with inhibitory ligands such as PCNA (40).

## 3. Role Of T-Box Transcription Factors in Cell Phenotype and Function: Parallels in NK and T Cells

T-bet and Eomes are members of the T-box family of transcription factors which are characterised by the presence of a conserved T-box DNA binding domain, and have been evolutionary entrenched since the early metazoans (41). They are the only members of the T-box transcription factors expressed in the immune system and are fundamental to the development and existence of NK cells (42). The expression of T-bet and Eomes are tightly regulated throughout the different stages of NK cell development (43–45), with the presence of both transcription factors being crucial for proper development. NK cells fail to develop in the absence of both transcription factors, and deletion of either T-bet or Eomes results in NK cells that are unable to reach full maturation (45). Their expression is also deemed to be crucial for mature NK cell cytotoxic function (42), with NK cells from Eomes knockout mice demonstrating reduced IFNγ production, as well as Eomes^+^ cells producing more perforin than Eomes^-^ cells (45). T-bet has also been shown to bind to the promoters of perforin (*PRF*), granzyme B (*GZMB*) and IFNγ (*IFNG*) (43), suggesting a role for T-bet in regulating NK cell cytotoxicity. Together, these studies suggest that changes in T-bet and Eomes expression and activity may potentially be a surrogate for NK cell dysfunction.

T-bet and Eomes are also key transcription factors in T cells, regulating both function and homeostasis (46, 47). They have both been demonstrated to be essential for effective T cell antitumour response (48), with dysregulation in expression levels being linked to an exhausted T cell phenotype in various contexts including infection and cancer (49, 50). Eomes expression in exhausted T cells correlates with severity of exhaustion during chronic viral infection, along with high inhibitory receptor expression (51). Additionally, Eomes^+^T-bet^low^ CD8 T cells exhibit functional impairment in leukaemia patients, partly attributable to Eomes binding to the promoter of the inhibitory receptor TIGIT and positively regulating its expression (52). Interestingly, a recent study reported that T cell exhaustion may not simply be defined by the overall T-bet and Eomes expression, but rather that high nuclear Eomes:T-bet ratio is key in defining T cell exhaustion in both chronic LCMV infection as well as in human melanoma patients (53). This provides further insights into the biology of how these T-box transcription factors regulate T cell exhaustion.

However, whether an altered balance of T-bet and Eomes affects NK cell function and exhaustion in cancer, and whether dysfunctional NK cells show a similar expression pattern of T-bet and Eomes compared to that of exhausted T cells, remain less clear. Circulating healthy human NK cells were reported to express high T-bet and low Eomes, and an intricate balance between T-bet and Eomes seems to characterise NK cell function (54). In the context of cancer, a study in 2012 reported a murine model of cancer where loss of NK effector functions was accompanied by a rapid downregulation of both T-bet and Eomes (55). In another study involving exhausted murine NK cells characterised by high expression of inhibitory receptors, TIM-3 and PD-1, reduced Eomes expression was similarly observed, although this was also accompanied by increased T-bet (56). Studies on melanoma patients showed that both T-bet and Eomes were downregulated in NK cells (31). However, in lung cancer patients, only reduced Eomes expression was found to correlate with cancer progression and NK cell dysfunction (57). Furthermore, overexpression of Eomes in NK cells (55) or adoptive transfer of Eomes^hi^ NK cells (57) was able to reduce tumour burden in mice (55). These reports suggest that beyond NK cell surface receptor expression, altered expression of both T-box transcription factors and their corresponding transcriptional programs also play a role in mediating NK cell cytotoxicity against cancer, and could be used to assess NK cell dysfunction. Although the role of T-bet in mediating NK cell function against cancer remains less clear, increased Eomes expression appears to be consistently implicated in improved antitumour activities of NK cells, both in human and murine models.

Reports have suggested that T-bet and Eomes have cooperative or partially redundant functions (58), given that they both share a homologous DNA binding sequence (41). However, their specific contributions and mechanisms in regulating NK cell function, exhaustion and/or activation await further studies and elucidation. Interestingly, recent work confirms that while T-bet and Eomes do indeed compete for the same T-box consensus sequence, increasing the concentration of one transcription factor can displace and reduce binding of the other to the same consensus sequence (53). Additionally, binding of T-bet or Eomes results in differential transcriptional control. For example, in T cells, T-bet is a strong repressor of PD-1 (59), however, Eomes binding to the same T-box sequence upstream of the PD-1 gene results in a much weaker repressive activity (53). Whether or not such a mechanism is relevant to NK cells and whether it could be a contributor to NK dysfunction is still unknown.

In recent years, it has been reported that NK cells can be converted to ILC1-like cells, which display poorer cytotoxic capabilities (60), as evident from reduced control over tumour burden and metastasis (61, 62). Distinct from NK cells, the ILC1s do not express Eomes (60), further implicating this T-box transcription factor in marking functional and mature NK cells rather than its less cytotoxic ILC1 counterpart. It is also possible that previous studies showing the downregulation of Eomes (31, 55) and the corresponding reduction in NK cell function could have been the result of NK cells acquiring an ILC1-like phenotype, although this remains to be further proven. Early studies initially used CD49a and CD49b to distinguish between both cell types, with NK cells characterised as CD49a^-^CD49b^+^ and ILC1s as CD49a^+^CD49b^-^ (60). However other groups have also reported the expression of CD49a^+^ NK cells in the liver as well as in lung and blood (63, 64), thus complicating the use of these two markers in distinguishing between NK cells and ILC1s. Much subsequent data also suggested the difficulty and lack of any distinct marker in delineating ILC1s from NK cells, due to overlapping phenotypes in different tissues and contaminating cell types based on gating strategies (65). Despite the difficulty in differentiating between NK cells and ILC1s through the use of CD49a/b, it is perhaps still worth noting that peripheral NK cells from prostate cancer patients also express CD49a, along with high levels of the inhibitory receptors PD-1 and TIM-3, and exhibit impaired degranulation (66). Tumour-infiltrating NK cells with poor cytotoxicity and upregulated inhibitory receptors were also found to express CD49a in hepatocellular carcinoma patients (67, 68). Hence, regardless of whether or not the observed cell subsets are indeed NK or actually ILC1, these studies suggest that CD49a could possibly be used as a marker of dysfunctional NK cells. Therefore, it might be necessary and useful to study other possible markers of ILC1s, for example CD127 and CD69, although these markers should still be used with caution as they appear to be differentially expressed in ILC1s in various tissues (69, 70). Further exploration of this controversy and insights into the plasticity of the NK cells and ILC1s could provide new perspectives on how the pro-tumorigenic factors and physicochemical conditions in the tumour microenvironment (TME) might shape the NK cell response.

**Figure 1:**
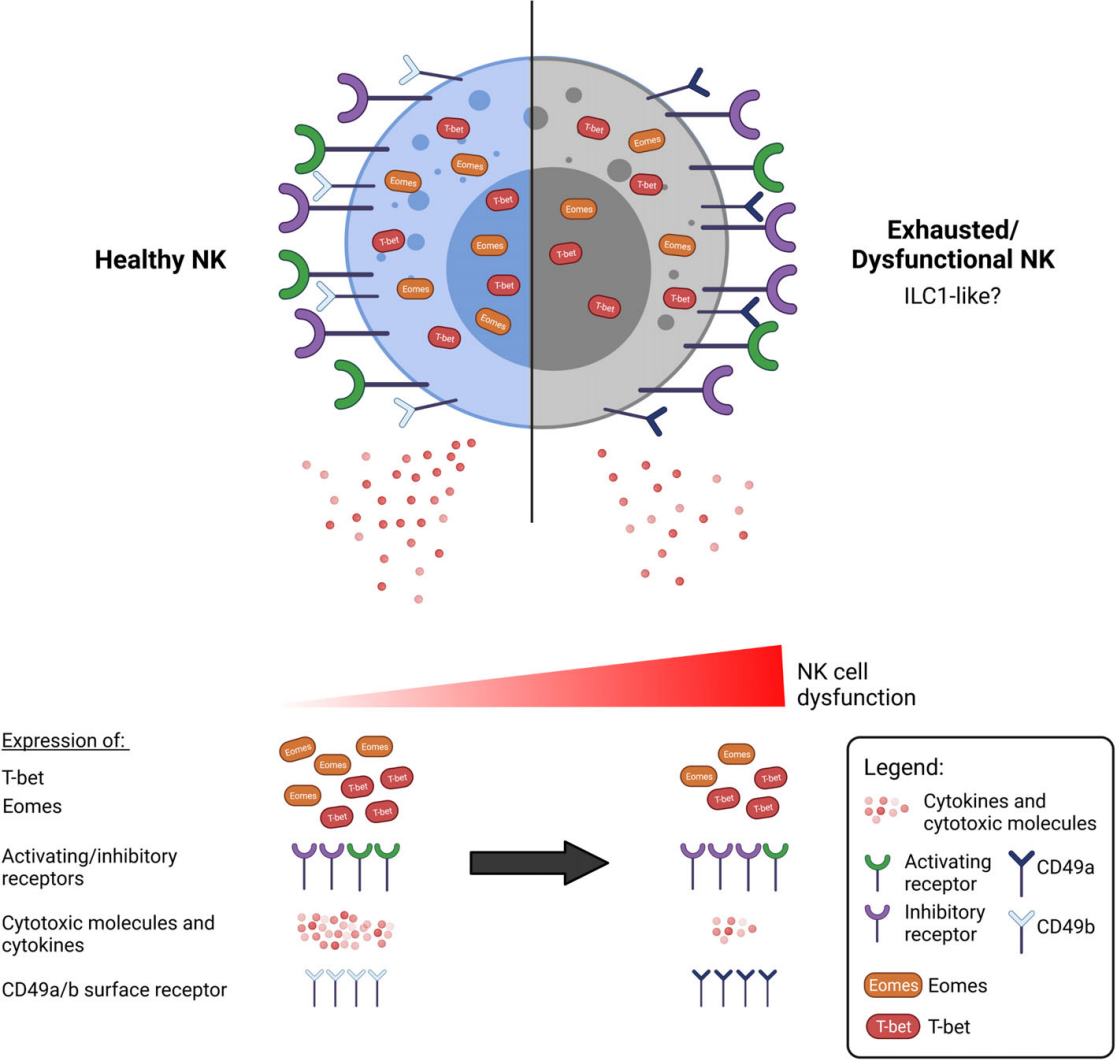
A dysfunctional or exhausted NK cell may be characterised by more than an alteration of surface receptor expression. Functionally impaired NK cells are commonly observed in cancer to exhibit reduced anti-tumour activity, including secretion of cytokines and cytotoxic molecules. There is currently a lack of consensus as to what constitutes an exhausted NK cell. NK cell dysfunction exists over a spectrum, and various aspects that may define exhaustion have been researched thus far. Beyond the well-discussed upregulation of inhibitory receptors such as TIGIT, NKG2A, and downregulation of activating receptors such as NKG2D, DNAM-1, we propose that the exhausted NK cell can also be marked by the altered expression of the T-box transcription factors, T-bet and Eomes. Additionally, exhausted NK cells exhibit a phenotype resembling that of the less cytotoxic ILC1 cells, which are additionally marked by the expression of CD49a.

In summary, we propose that the characterisation of dysfunctional NK cells goes beyond functional impairment and an altered surface receptor phenotype. An all-encompassing definition for NK cell exhaustion may thus include the expression of surface markers such as CD49a/b as well as expression of key T-box transcription factors (**Figure 1**), although this should be studied in greater detail in the context of NK cells and in cancer.

## 4. Context-Specific Mechanisms Driving Altered NK Cell Phenotypes in the Tumour Microenvironment

The altered surface receptor expression and transcriptional programs observed in cancer-associated NK cells are known to perturb the downstream signalling programs mediated by these receptors, such as the proinflammatory DNA-PKCs-Akt-NF-*κ*B, MAPK, AKT and ERK signalling pathways (19). The proper regulation of these signalling cascades in healthy NK cells drives appropriate NK cell gene expression, target cell conjugation, degranulation, cytokine release and cytotoxicity (19). The loss of NK cell function has been shown to occur progressively, correlating with disease severity in cancers (71, 72). Since the functions of NK cells are regulated by signals from activating and inhibitory receptors, with many of these receptors converging onto the same pathways regulating NK cell cytotoxicity [as mentioned (19)], it is thus likely that any alteration in signalling will drive progressive impairment of NK cell function. The restoration of NK cell function and cytotoxicity upon targeting these receptors through blockade or overexpression (**Table 1**) have also firmly established that these phenotypic differences play a crucial role in mediating NK effector functions. Despite such attempts at reviving NK functions, the lack of a singular consistent exhausted phenotype reported by various groups suggests that dysregulation of NK cell function is likely modulated in a tumour- and context-specific manner. The heterogeneity in the TME in different cancer types, as well as between individuals harbouring the same cancer would demand an array of NK phenotypes to cater to varying contexts. Thus, while strategies to restore NK to a healthy phenotype are poised as promising new avenues to be further explored for cancer immunotherapy, it is imperative to understand the specific mechanisms in the TME contributing to such phenotypic and functional alterations.

### 4.1 Loss of Tumour Cell Immunogenicity Prevents NK Cells From Recognizing Cancer Cells

Tumour progression is associated with the presence of a TME that is tolerant and immunosuppressive, which results in the evasion of cancer cells from immune surveillance. The heterogeneity of tumour cells in the TME such as differential ligand profile and secreted factors (73), further contributes to the downregulation of NK cell function against cancer. NK cell engagement with target cells and ligand-receptor interaction are thought to be the first steps leading to the formation of the immune synapse (contact point between cancer cell and NK cell) and subsequent activation of NK cytolytic capability (74). Therefore, it is deemed that the mechanisms of immune escape by cancer cells would occur largely through the modification of cancer cell ligands or NK surface receptors, or both.

The downregulation of activating ligands and upregulation of inhibitory ligands in immune responsive cells are associated with tumour progression, which is accompanied by a loss of tumour immunogenicity. Thus, the increased engagement with inhibitory ligands, or lack of engagement with activating ligands on cancer cells shifts the NK cell towards an inhibited state, resulting in failed tumour clearance. Some mechanisms underlying the alteration of ligand expression in tumour cells include the dysregulation of microRNA (miRNA), which has been observed in many cancers (75). miRNA dysregulation decreases the expression of activating ligands, MICA/B (76, 77) and ULBP2 (76), and increases the expression of inhibitory ligands, PD-L1 (78) and MHC class I molecules (79, 80) on tumour cells, which can limit the immunosurveillance capabilities of NK cells. Furthermore, post-translational modifications such as ubiquitination and SUMOylation of ligands expressed in cancer cells can alter the localization and activities of the ligands (81). For example, downregulation of PVR, the activating ligand for the receptor DNAM-1, is attributable to its SUMOylation in multiple myeloma, resulting in its intracellular localisation (82). In Kaposi’s sarcoma-associated herpesvirus infection, MICA, which is a ligand for NKG2D activating receptor, undergoes ubiquitination, which prevents its cell surface localization (83). The observation of similar intracellular compartmentalisation and regulation occurring with the NK cell ligand MICA in Kaposi’s sarcoma (83) shows that such a mechanism of regulation may be generalizable across different disease contexts. These observations (e.g., ubiquitination of NK receptor ligands) also increase the possibility that the expression and compartmentalisation of other ligands for NK cell receptors may be similarly regulated through different mechanisms of post-translational modifications.

The above-mentioned mechanisms are non-exhaustive and, on the whole, traditionally enable tumour escape from NK immunosurveillance through shifting the balance of ligand expression towards generating a decreased activating signal in NK cells. However, high expression of activating ligands has been observed in some tumours, although this causes the unexpected downregulation of the corresponding NK cell receptors, leading to NK cell dysfunction instead of augmenting tumour surveillance. For example, the high and sustained expression of NKG2D ligands on many tumours results in downregulation of NK cell NKG2D receptor through endocytosis (84, 85), which is believed to be a feedback mechanism regulating NK cell tolerance (86). Current anti-cancer chemotherapy regimens may also cause the upregulation of NKG2D ligands (87, 88), and while this may transiently upregulate NK cytotoxicity, a sustained and prolonged period of high ligand expression for cancer immunotherapy could exhaust NK cytotoxicity and pose an area of concern for long-term efficacy of NK cell function. The recognition of tumour cells and subsequent regulation of NK cell activity is thus not simply based on a straightforward interaction of a receptor with its cognate ligand leading to receptor activation. Thus, the robustness of NK cell phenotype in response to cancer cells are important considerations in the design of future therapeutic strategies targeting NK cell receptors.

### 4.2. Downregulation of Intrinsic NK Cell Function by Factors in the TME

Apart from a lack of tumour immunogenicity, soluble factors in the TME may also alter intrinsic NK cell function, thus further contributing to poor NK cell antitumour activity. For example, this can occur through the regulation of NK cell surface receptors, NK cell gene expression and metabolism, and will be discussed in this section.

The well-studied immunosuppressive cytokine, TGF-β, is key for cancer progression and it is known to elicit responses from stromal cells in the TME (89). Various cancer types secrete high levels of TGF-β with wide-ranging effects on NK cells in the TME. Recent studies indicate that a high level of TGF-β in the TME further promotes expression of TGF-β receptors on NK cells resulting in a positive feedback loop (90). Prolonged exposure to TGF-β significantly downregulates expression of NK cell activating receptors NKG2D, CD16, and NKp30, while upregulating the death ligand FasL and inhibitory receptor NKG2A in healthy donor NK cells (91), which dulls the recognition of target cells. This regulation of NK cell receptors by TGF-β has been shown to occur *via* various miRNA pathways (92). Additionally, TGF-β reduces T-bet expression through SMAD3 signalling, resulting in decreased expression of its target gene *IFNγ* (93), which would contribute to a reduction in NK cell function. Indeed, upon continuous stimulation with TGF-β, NK cells from healthy donors demonstrated reduced cytotoxicity against osteosarcoma cell lines, despite increased degranulation (91). Such chronic exposure to TGF-β is likely to be an accurate representation of TGF-β secretion by cancer cells in the TME.

The mammalian target of rapamycin (mTOR) forms two complexes known as mTORC1 and mTORC2. While mTORC1 integrates signal from growth factors and metabolism, mTORC2 is mainly involved in growth and proliferation (94). In NK cells, it was demonstrated that priming by dsRNA mimetic poly (I:C) or IL15 activates mTORC1 and 2, which results in enhanced metabolism (95). The enhanced metabolism in NK cells characterised by enhanced glycolysis correlates positively with the production of cytolytic molecules such as granzyme B (96). Interestingly, TGF-β in the tumour microenvironment is able to cross-talk with mTOR signalling pathway. Recently, TGF-β has been shown to induce early inhibition of mTOR activity in NK cells by opposing the phosphorylation of mTOR substrates S6, 4EBP1 and AKT. Furthermore, the inhibitory effects of TGF-β were comparable to the mTOR inhibitor rapamycin, thereby limiting metabolic activities in activated NK cells (97). This was one of the few studies that directly investigated the molecular mechanism of TGF-β inhibition on NK cells. However, there are still discrepancies on whether TGF-β signalling and mTOR inhibition (e.g. by rapamycin) converges. For instance, TGF-β induces TRAIL expression while mTOR inhibition does otherwise (97). Furthermore, it was previously demonstrated in other cell types that TGF-β/Smad3 signalling activates mTOR to promote collagen production by increasing HIF-1α expression (98). Hence, it is possible that there exists an unknown scaffold protein that spatio-temporally regulates TGF-β inhibition of mTOR in NK cells.

Other than TGF-β, tumour cells also secrete many other immunosuppressive factors, for example, PGE2, PCLP1, IDO, which have been found to inhibit NK cell function and downregulate activating receptor expression (99, 100). However, unlike TGF-β, the mechanisms through which these cytokines function to regulate NK cells, and whether and how they drive other phenotypic changes beyond altered receptor expression, are unclear and still await further studies. A more detailed review on these soluble factors in the TME, and their suppressive effects on NK immunosurveillance capabilities has been provided by Melaiu et al. (101).

### 4.3 Inhibition of NK Cell Cytotoxicity by Other Immune Cells in the TME

**Figure 2:**
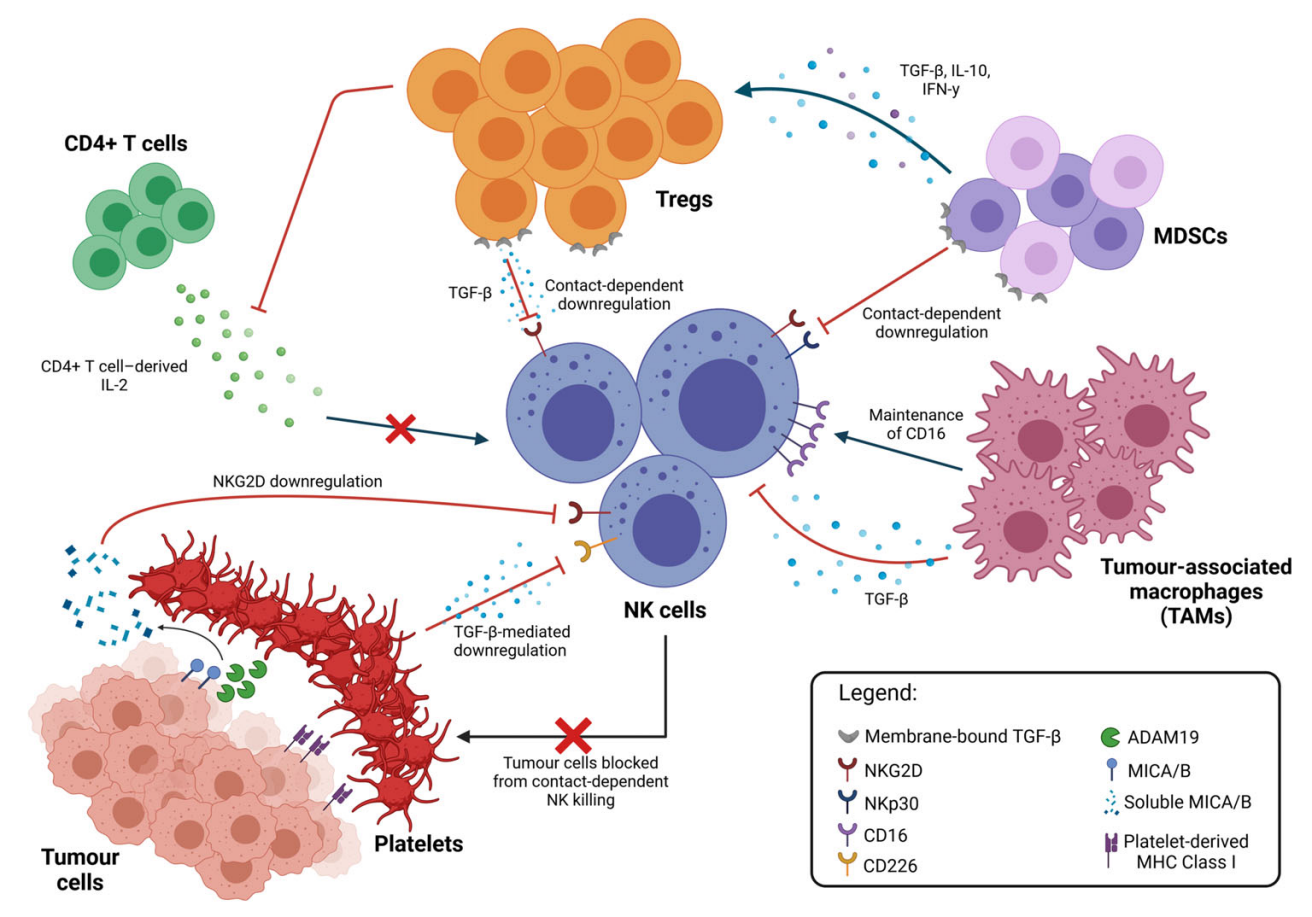
Inhibition of NK cell cytotoxicity by other immune cells. Immune suppressive cell types in the TME interact directly with NK cells to inhibit their function through both contact-dependent and contact-independent downregulation of NK cell activating receptors, typically mediated by TGF-β. TAMs promote the maintenance of membrane-bound CD16, which reduces NK activation. NK cell function can also be modulated indirectly through multiple interactions between these different cell types. Cytokine secretion by MDSCs promote the expansion of Tregs, which apart from directly inhibiting NK cell functions, also limits the availability of IL-2 from CD4+ T cells, thus preventing NK cell stimulation. Platelets in the TME also interact with tumour cells directly, shielding them from NK cell recognition and killing; cleaving activating ligands on the tumour cell surface; and equipping tumour cells with MHC Class I molecules to facilitate immune evasion.

The TME harbours a diverse repertoire of immune cells from innate and adaptive immune systems. Various soluble factors produced by these cells and cross-talks between them are a major contributor towards the suppressive conditions that allow for tumour progression and the impairment of NK cell function (**Figure 2**).

Macrophages are typically categorised into the classical M1 or the alternatively activated M2 macrophages, and this differential polarisation occurs as a result of different activation stimuli (102). M1 macrophages secrete pro-inflammatory cytokines and kill pathogens, whereas M2 macrophages are anti-inflammatory and tumour-promoting (103). Tumour-associated macrophages (TAMs) make up the majority of immune cells found in the tumour site (104). Unsurprisingly, TAMs express an M2-like phenotype in solid cancers, and large numbers of M2 TAMs correlate with poorer prognosis (105). M2 TAMs inhibit NK cell function *via* the secretion of immunosuppressive TGF-β (refer to section 4.2 for details) and in a contact-dependent manner, to reduce the production of IFNγ and TNFα (106, 107). Additionally, it has also been proposed that under specific chronic inflammatory conditions of cancer, TAMs protect tumour cells from NK cytotoxicity by preventing the shedding of membrane-bound CD16 (108). CD16 is an Fc receptor whose shedding indicates NK cell activation and increases NK engagement with target cells (109). While the maintenance of surface CD16 indicates inhibition of NK cell function, it is worth noting that simultaneously, CD16 acts as an important activating receptor in antibody-dependent cytotoxicity, and it is a well-explored therapeutic target (110). Engagement of CD16 with therapeutic antibodies has been shown to improve NK cell response to restimulation and result in higher cytotoxicity towards tumour cells (111). Thus, despite being a seemingly tumour-protective mechanism, the maintenance of CD16 on NK cells by TAMs may instead present a window of opportunity when viewed from a therapeutic intervention perspective.

Myeloid-derived suppressor cells (MDSCs) consist of progenitors and precursors of myeloid cells (112), which are found in significantly higher numbers throughout tumour progression (113). MDSCs express membrane-bound TGF-β, which has been found to impair NK cell functions (113). *In vitro*, coculture with NK cells revealed a downregulation of NK cell cytotoxicity through STAT5-mediated activity (114). Interaction between MDSC and NK cells also downregulates NK cell receptor, NKp30, and blockade of NKp30 relieves the MDSC-induced inhibitory effects on NK cells (115). These observations suggest that MDSCs express ligands that inhibit NK cell functions through a cell-cell contact-dependent manner. Although generally accepted to be immunosuppressive, it appears that whether MDSCs function to activate or inhibit NK function, is context-dependent. Indeed, another study identified RAE-1 expression on MDSCs to induce NK activation in part through the NKG2D receptor (116). Furthermore, a plethora of cell types and soluble factors *in vivo* also contributes to the complexity of NK activation.

Regulatory T cells (Tregs) are the immunosuppressive subset of CD4^+^ T cells, and their upregulation is typically correlated with poor prognosis and cancer progression (117). In healthy individuals, crosstalk between Tregs and NK cells is an important self-tolerance mechanism (118). However, in cancer patients, elevated Treg levels are often found to correlate with low frequency and poor function of NK cells (119), suggesting a disrupted Treg-NK cell-cell balance or interaction. Tregs have been found to limit the availability of CD4^+^ T cell-derived IL-2, which is required to stimulate NK cells (120). Similar to MDSCs, membrane-bound TGF-β on Tregs was also suggested to suppress NK cell cytotoxicity and downregulate the expression of activating receptor, NKG2D (121, 122). However, the inhibitory effects of Treg membrane-bound TGF-β on NK cells appear to be distinct from the effects of MDSC membrane-bound TGF-β (113), as discussed above (section 4.2). This suggests important distinctions between the mechanisms contributing to NK cell exhaustion by different immune cell types in the TME.

Platelets have also been increasingly recognised as another key player contributing to cancer progression and metastasis (123). The inhibition of NK cell function by platelets was first discovered to be through providing a physical barrier to tumour cells, directly protecting them from contact-dependent NK killing (124). Beyond a physical barrier, platelet cloaking also modulates the expression of NK cell receptors and corresponding tumour cell ligands (125). For example, they promote the loss of the NKG2D ligands like MICA/B on tumour cells, possibly through upregulating the expression of ADAM19, a protease responsible for MICA/B cleavage (126). The release of MICA/B into the TME further inhibits NK cell activity, since soluble NKG2D ligands are known to suppress NKG2D receptor expression on NK cells (127). Downregulation of other NK cell activating receptor ligands, CD112 and CD155, was also observed in platelet-cloaked breast cancer cells, along with their corresponding activating receptor, CD226 on NK cells (126). This was further shown to be attributable to the presence of platelet-derived TGF-β (126). Interaction and contact between platelets and tumour cells also promotes the transfer of platelet MHC class I molecules to tumour cells *via* trogocytosis (128). This facilitates tumour evasion of immune response as tumour cells are no longer recognised *via* the ‘missing self’ mechanism. Additionally, like all other cell types in the TME, platelets release a large variety of chemokines and soluble factors, which can serve to recruit other adaptive and innate immune cells to the tumour site (129), many of which interplay with NK cells with potentials for immunomodulating NK cytolytic functions.

### 4.4 Altered Cytoskeletal Dynamics in Both NK and Cancer Cells – A *‘Tug-of-War’*

As essential players in initiating and regulating NK cell function, NK cell surface receptors and their corresponding ligands have understandably been the subject of intense scrutiny and research. Here, we highlight the often-overlooked roles of the cell cytoskeletal network in the regulation of NK cell effector function, which arguably, is largely accountable for NK cell dysfunction in the TME.

The cell cytoskeletal network consists of long filamentous components represented by the microtubule and actin. Apart from modulating and organising the internal architecture of the cell, these cytoskeletal components along with their accessory proteins are instrumental in the generation of intracellular forces for mechanotransduction and signalling. The coordinated efforts of NK cytoskeleton in response to transformed cancer cells serves at least three functions: (i) the formation and organization of immune synapse for lytic granule secretion, (ii) the establishment of cell polarity through microtubule-organizing centre (MTOC) and (iii) the modulation of NK receptor nanoscale organization and interaction with tumour ligands. These processes amplify into a multitude of indispensable arsenal for NK cells to fight cancer cells and will be reviewed in more detail in the following sections.

#### 4.4.1 Microtubules – Beyond a Roadmap for Granule Trafficking

The microtubules are helical lattices that are assembled from repeating units of polymerized α and β tubulin monomers. They originate from the MTOC, which consists of the centrosomes and pericentriolar material. In eukaryotic cells, microtubules function as an architectural framework that augments structural organization of internal organelles (e.g., the nucleus and cytoskeletal components). In addition, they are the internal transport network which motor proteins such as kinesin and dynein traffic along. In the context of NK cells, much has been focused on how MTOC organization and orientation regulates NK cytotoxicity.

The reorientation of MTOC to the immune synapse is a rapid event that may occur as quickly as 5 minutes after receptor engagement/stimulation (130). Unfortunately, there is a lack of mechanistic understanding of MTOC orientation in NK cell response to cancer cells. Limited data suggests that activating receptors (e.g. NKG2D, NKp46 and NKp30) on NK cells activate two non-redundant signalling pathways, the PI3K → ERK2 and the PLCγ → JNK1 pathways for MTOC polarization (131). The integrin, LFA-1 on NK cells was also identified to facilitate the adhesion of NK to target cells and mediate polarization of lytic granules containing perforin through the engagement of ICAM-1 ligand expressed in target cells (132). However, NK cells in the TME often lack or downregulate one or more activating receptors, and whether there exists receptor redundancy for MTOC polarization, remains poorly defined. Interestingly, the overexpression of ICAM-1 is observed in several cancer cells e.g. breast and lung cancers (133, 134). Hence, it has been construed that LFA-1 expression on NK cells is the main driver of MTOC polarization during NK-antitumor immunosurveillance. Subsequently, it was found that the accumulation of PKCϵ, PKCη, and PKCθ in NK cells precedes MTOC reorientation since siRNA knockdown of these proteins disrupted MTOC reorientation (135). Simultaneously, lytic granules move towards the MTOC with the help of the motor protein dynein to facilitate the convergence of lytic granules to the immune synapse (136). The exact motor protein that lytic molecules traffic along the microtubule is under debate. In the context of NK-cancer immunomodulation, much less is known on whether and how NK-resistant cancer cells can affect microtubule function, viz whether cancer cells affect lytic granule trafficking, convergence and/or MTOC polarization. A recent study provided evidence of defective granule polarization (but not granule clustering) in NK-92 cells upon encountering NK-resistant breast cancer cells (137). These findings in part suggest and support previous reports that, (i) granule convergence/clustering precedes MTOC polarization towards the immune synapse in the context of NK-cancer immunosurveillance and (ii) cancer cells immunomodulate NK signalling by the perturbation of MTOC orientation signals (e.g. PLCγ → JNK1 pathways). Indeed, the PLCγ pathway that promotes MTOC polarization was shown to be triggered by NK activating receptors (131), which are often downregulated in the tumour microenvironment.

#### 4.4.2 Actin Dynamics for NK Cytotoxicity

The actin network provides the structural basis for immune synapse formation and function. Along with actin associated proteins (e.g. ARP2/3, WASp, Wave1 Cdc42 etc.), the actin complexes are the major drivers of NK cell effector function against foreign entities (138). Hence, it is unsurprising that proper regulation of actin polymerization, dynamics and organization is key for the formation of the immune synapse and subsequent release of cytotoxic molecules, as well as for promoting NK cell motility and effective migration and infiltration into tumour sites (136).

*De novo* actin accumulation and rearrangement at the immune synapse marks the start of the effector stage of NK degranulation and this is a critical step towards eliciting an effective cytotoxic response. Indeed, actin targeting drugs such as cytochalasin (139), lantrunculin (140) or jasplakinolide (141) limit NK cytotoxicity. A study utilizing live-cell super-resolution microscopy overcame the spatio-temporal resolution limitations of NK cells (142) and demonstrated that the stochastic clearance, formation and disappearance of filamentous actin (F-actin) at the immune synapse is critical for size permissive degranulation of cytolytic components (142). In addition, various actin associated proteins were shown to be important for NK lytic function. For instance, the hematopoietic cell-specific action associated protein coronin 1A colocalizes with F-actin at the immune synapse and regulates F-actin density for size permissive granule penetrance to the membrane (143). The non-muscle myosin IIa generates contractility and is also important for degranulation (144). Dysregulation of contractility did not affect lytic granule conjugation but increased actin density at the plasma membrane and severely limited degranulation (142). There are more than 100 actin associated proteins that regulate NK cytotoxicity against cancer cells, which are beyond the scope of this review. A detailed review on these actin associated proteins and how they affect NK immune synapse formation and function can be found in a recent review by Ben-Shmuel et al. (136).

#### 4.4.3 Spatio-Temporal Cytoskeletal Co-Ordination of NK Cytolysis

There is limited information based on NK-cancer co-culture models or *in vivo* research on how cancer cells can directly affect NK immune synapse and actin dynamics. A recent study suggested that activating signals at the immune synapse promote fast actin retrograde flow and reduces β-actin, SHP-1 interaction, thereby enhancing NK cytotoxicity (138). In addition, actin polymerisation has been established to be a downstream event of NK activating receptor signalling (145), which is often dysregulated in cancers. Wilton et.al., showed that the actin regulatory protein Ena/VASP-like (EVL) is recruited to the immune synapse by NKG2D-DAP10 complex and involves the DAP10-dependent Grb2-VAV1 associated pathway (145). Looking forward, there are evidences suggesting that actin-mediated intracellular contractility could alter nuclear mechanotransduction (146), which results in chromatin remodelling and impacts the intracellular localisation of transcription factor co-activators (147). If applied to the T-box transcription factors, the actomyosin based contractility could be conversely altered with implications on the expression and localisation patterns of T-bet and Eomes, which are key in regulating NK cell functions and their antitumour activities, as discussed above (Section 3). In addition, since the TME is known to be hypertonic (148), there is an underappreciated link to how the TME regulates NK cell function through altering its contractility, independent of NK surface receptors.

In cancer cells, actin remodelling has also been found to aid evasion from NK killing. For example, accumulation of actin at the immune synapse in breast cancer cells encountering NK limited the accumulation of the cytotoxic molecule granzyme B in the cancer cells, protecting them from lysis and resulting in lowered apoptosis of the tumour cell (149). The cancer cell lines used in this study (149) exhibited different levels of actin accumulation at the immune synapse, and it was suggested that compared to epithelial-like cancer cells, mesenchymal-like cancer cells had a higher capacity to generate actin accumulation at the immune synapse, thus contributing to differential and greater resistance to NK killing. The cellular cytoskeletal dynamics on both sides of the immune synapse, which influences the contact-dependent nature of NK-mediated killing, appears to play a pivotal role in the success or failure of NK cell elimination of target cells. This can occur as a result of factors inherent to the cancer type itself, but may also be attributable to the hypertonicity of the TME (148) which can induce cellular contractility.

Thus far, many studies have focused on the functional consequences of cytoskeleton dynamics in regulating NK cell function. It is therefore pertinent for future studies to unravel the molecular and biophysical mechanisms that regulate a dynamic cytoskeletal network for NK cytotoxicity against cancer cells. For instance, to ask whether scaffold proteins exist to spatio-temporally coordinate lytic granule trafficking, MTOC polarization and actin dynamics at the immune synapse. Such information will be helpful for understanding whether and how NK exhaustion can be phenotypically characterised by altered cytoskeletal components. Furthermore, *ex vivo* perturbation of the cytoskeletal network in NK cells may hold promising future therapeutic opportunities.

## 5. Looking Beyond Targeting NK Cell Receptors for Therapeutic Advances

Many studies have thus far focused on elucidating the effects of individual TME players on NK cell function, which has provided valuable insights into the various mechanisms underlying NK cell exhaustion. As reviewed herein, these mechanisms tend to converge on the alteration of NK cell surface receptors, hence much emphasis is currently placed on targeting of these surface receptors in an effort to restore NK cell function. However, given the large number of NK cell receptors, the complexity of the TME and the numerous players exerting their inhibitory effects on NK cells *via* multiple receptors, it is challenging for current therapies targeting specific receptors to contend with the inhibitory effects of the TME *in vivo*. Therefore, along with current developments in targeting NK cell receptors, alternative approaches for the revitalisation of NK cells should also be taken into consideration. For instance, the T-box transcription factors in NK cells have been shown to be key for NK cell function and development, and several reports thus far have suggested their expression levels to be dysregulated in exhausted NK cells. Given that significant progress has been made in developing drugs to target transcription factors (150), further study on how these transcription factors are precisely regulated by the TME may open up a new avenue of treatment to restore NK cells to their full cytotoxic capacity. To this end, whether and how transcriptional signals can be desensitized for ‘self-tolerance’ in response to persistent stimulation by cancer cells, could be addressed. Additionally, an increasing interest and understanding on how cytoskeletal dynamics shape NK cell cytotoxicity is anticipated to shed new insight into the regulation of NK cell function through its impact on transcription factor expression, receptor expression, and/or other yet to be elucidated mechanisms. The integration of these areas of knowledge will be key to the advancement of novel therapies that will holistically target multiple aspects of the exhausted NK cell.

The use of CAR-NK cells in immunotherapy has yielded optimistic results, and provides several advantages over CAR-T cells, such as improved tolerance, reduced toxicity as well as multiple sources from which NK cells can be derived (151). However, CAR-NK cells are still associated with various technical and biological limitations, such as difficulties in CAR delivery into NK cells, the short lifespan of NK cells in the absence of additional cytokine treatment, the requirement for *ex vivo* expansion prior to infusion, low tumour infiltration capacity and reduced cytotoxicity *in vivo* (151, 152). While strategies to engineer NK cells for improved performance are being studied, other areas of therapy are now also being explored for potential use in combination with established cancer therapy. One such area of interest is the use of nanomaterials such as liposomes and nano-emulsions to enhance NK cell activation as well as to modify the immunosuppressive TME (153). Additionally, other studies are also looking beyond the traditional checkpoint inhibition targets of NK surface receptors, towards proteins such CEACAM, chemokine receptors and even growth factors found in the TME; drugs targeting these molecules are already in early stage clinical trials (154). When used in tandem with existing NK cell-based therapy and other immune cell-based therapy or chemotherapy, there is greater hope that these emerging treatment options will provide increased synergy and efficacy, moving us towards achieving greater success in exploiting NK cells for cancer treatment.

Furthermore, recent advances in single cell studies involving genotyping and phenotyping have identified various NK cell phenotypes in the body (155, 156). These studies highlight the functional plasticity of NK cells, which results in a blurred boundary between ‘young’, ‘mature’ and ‘exhausted’ NK cells. For example, NK cells in the lungs have a more mature phenotype compared to those in other tissues. In mice, lung NK cells express high levels of mature markers NKp46, CD49b, CD11b, and Ly49 receptors (157). Systemic therapeutic interventions disregard the diverse subset(s) of NK cells (e.g. periphery NK cells) and this could affect premature upregulation and/or downregulation of NK cell activities. Moreover, the jury is still out on whether and how NK cells can interchange their phenotype to adapt to a more effective killer phenotype in different tissues. Hence, future precision-guided medicine should be open to embrace *omics* to nail down a targeted approach for NK-cancer immunomodulation.

**Figure 3:**
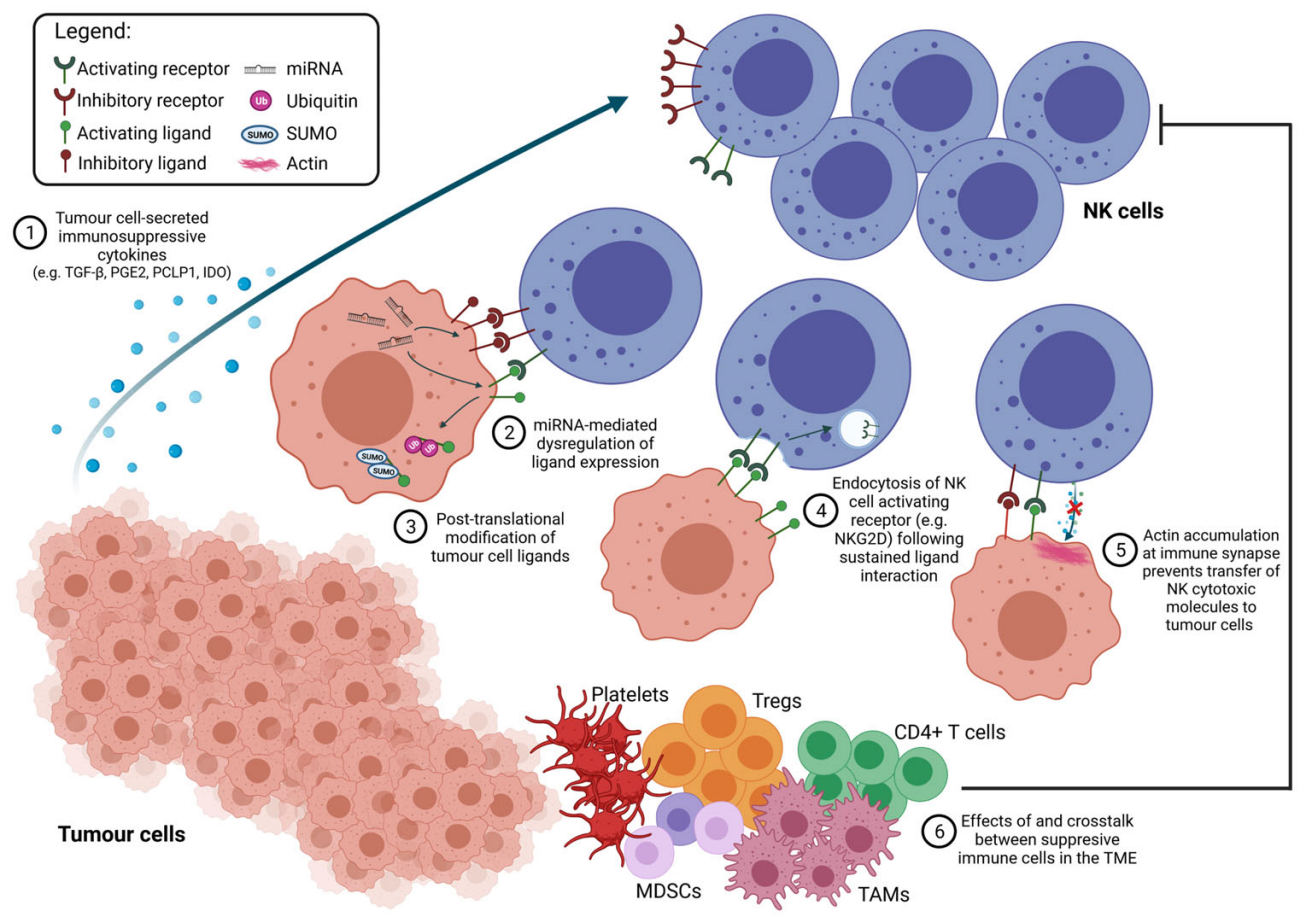
TME factors suppress NK cell function. Tumour-intrinsic properties play a significant role in the downregulation of NK cytotoxicity through altering NK-cancer receptor-ligand interaction, which shifts the NK cell towards an inhibited/exhausted state. (1) The secretion of immunosuppressive cytokines modulates NK cell surface receptor expression. (2) Dysregulation of miRNA promotes the upregulation of inhibitory ligands and downregulation of activating ligands. (3) Upregulated post-translational modification processes (e.g., ubiquitination and SUMOylation) in tumour cells cause intracellular localisation of activating ligands, allowing tumour escape from NK cell recognition. (4) Chronic exposure to activating ligands on tumour cells promotes a feedback mechanism leading to endocytosis of NK cell activating receptors. (5) Altered cytoskeletal dynamics in tumours also contribute to tumour cell resistance to NK cell killing. Additionally, effective recognition of cancer by NK cells is hampered by (6) the multitude of other immune cells in the TME which not only downregulate NK function, but also aid tumour cells in evading NK cell recognition.

This article has highlighted the uncertainty in characterising NK cell dysfunction, as well as recent studies made to widen our understanding of NK exhaustion. However, we also note the complex cell-cell interaction and molecular crosstalks occurring in the TME that contribute to, and shape NK cell function in distinct and complementary ways. With these large number of factors at play, it is clear that successful cancer immunotherapy should involve the use of combinatorial treatments, targeting different players in the TME, while also being mindful of the crosstalks between the various cell types present (**Figure 3**).

## Author Contributions

All authors contributed to the article and approved the submitted version.

## Conflict of Interest

The authors declare that the research was conducted in the absence of any commercial or financial relationships that could be construed as a potential conflict of interest.

## Publisher’s Note

All claims expressed in this article are solely those of the authors and do not necessarily represent those of their affiliated organizations, or those of the publisher, the editors and the reviewers. Any product that may be evaluated in this article, or claim that may be made by its manufacturer, is not guaranteed or endorsed by the publisher.

## Footnotes

1 TIGIT blockade alone increases IFNγ production in circulating NK cells, but has to be used in combination with IL-15 to promote increased cytotoxicity in tumour infiltrating NK cells(30).

2 NKp44 is classified as an activating receptor, but can also have inhibitory effects when engaged with inhibitory ligands such as PCNA(157)
